# Trait impulsivity prospectively predicts lower methadone treatment retention: moderation by pain catastrophizing

**DOI:** 10.1186/s13722-026-00699-7

**Published:** 2026-07-10

**Authors:** Annabelle M. Belcher, Kristen R. Hamilton-Moseley, Ebonie Massey, Mark Yoon, Thomas O. Cole, Michael D. Wagner, Amy S. Billing, Aaron D. Greenblatt, Eric Weintraub, Eric D. Wish

**Affiliations:** 1 Division of Addiction Research and Treatment, Department of Psychiatry, University of Maryland School of Medicine, Baltimore, MD USA; 2 Department of Psychology, University of Maryland, College Park, MD USA; 3 Center for Substance Use, Addiction & Health Research, University of Maryland, College Park, MD USA

**Keywords:** Opioid use disorder, Trait impulsivity, Pain catastrophizing, Medications for OUD, Methadone, Treatment retention

## Abstract

**Background:**

Empirical evidence supports the use of methadone as the standard of care for opioid use disorder. However, low retention in methadone treatment is one of the largest impediments to treatment success. Although several psychological and personality traits have been associated with substance use disorder, little work has studied the impact of these individual patient factors on addiction treatment outcomes such as treatment retention.

**Objectives:**

To examine the association of methadone retention with trait impulsivity and pain catastrophizing.

**Methods:**

We conducted exploratory analyses of data obtained from a randomized clinical trial (ClinicalTrials NCT02941809; registration date: 10/19/2016). Participants were adult treatment-seeking women and men diagnosed with moderate to severe opioid use disorder referred for methadone treatment (*N* = 80; 61% male) at an opioid treatment program in Baltimore, Maryland. Scores on the Barratt Impulsivity Scale and the Pain Catastrophizing Scale were collected following treatment intake, and the total window for observation of treatment retention was 365 days. Retention in methadone treatment was treated as a dichotomous outcome (1/0) based on whether a participant had dropped out of treatment at any point in time or if they missed 30 consecutive days in a row.

**Results:**

Cox regression models revealed that: (i) higher impulsivity was associated with a higher risk of dropping out of methadone treatment (i.e. lower treatment retention)[Hazard Ratio (HR) = 1.04, 95% confidence interval (CI) = 1.001–1.008, *p* < .05), and (ii) pain catastrophizing moderated the effect of trait impulsivity on risk of dropping out of methadone treatment, such that the effect of trait impulsivity on risk of dropping out of treatment was greatest in participants with low pain catastrophizing (HR=0.995, *p* < .01). These findings remained significant after controlling for age and treatment group.

**Conclusions:**

These findings suggest that trait impulsivity is a significant risk factor for low methadone treatment retention, especially for persons with low pain catastrophizing.

**Trial registration:**

ClinicalTrials.gov: NCT02941809 (registration date: 10/19/2016).

## Background

Fueled by increasing availability of synthetic opioids (chiefly, fentanyl), the current public health emergency represented by opioid use disorder (OUD) continues to plague the U.S. Effective functional recovery is possible with treatment with medications for OUD [1], of which methadone treatment (MT) has the largest evidence base. MT has been associated with significant improvements in several outcomes, including decreased drug use and crime and increased positive health outcomes [2, 3]. Randomized controlled clinical trials repeatedly have shown better outcomes with longer periods of MT [4–6]—but six-month nationwide retention rates rarely exceed 50–65% [7–9]. Low retention highlights the need to better understand the individual differences—and critically, the psychological factors—that contribute to MT outcomes.

Quantifiable and easily measured, certain personality and psychological traits have been identified as factors that overlap with, and may render individuals susceptible to, substance use disorder [10–12]. One personality trait that has received much study as a vulnerability factor is self-reported trait impulsivity [13]. Broadly defined as a predisposition toward rapid, unplanned reactions to internal or external stimuli with little regard for the negative future consequences [14–16], high trait impulsiveness has been associated with addiction to several drugs, including opioids [17–19].

Trait impulsivity also may scale with opioid addiction severity, as opioid withdrawal and consumption are positively associated with higher levels of trait impulsivity [17]. This increased tendency to behave impulsively may contribute to the high treatment drop-out rate observed in OUD populations; but to our knowledge, the relationship between impulsivity and opioid addiction treatment retention has not been explicitly tested.

A separate psychological construct with relevance to addiction is pain catastrophizing, a mental set involving a cascade of negative thoughts and feelings that imposes on anticipated or actual painful experiences. Pain is interpreted as extremely threatening during pain catastrophizing [20], and this cognitive process shapes emotional, functional, and physiological responses to pain [21–23]. Pain catastrophizing has long been associated with higher severity for patients suffering from acute and chronic pain conditions [24–27]. Studies in opioid-prescribed individuals suggest that pain catastrophizing is associated with an increased liability for development of OUD—with pain catastrophizers using larger amounts of prescription opioids [28], reporting higher levels of craving [29], and misusing their opioid prescriptions at significantly greater rates than non- catastrophizers. An estimated 37–60% of patients being treated with methadone for OUD have chronic pain, suggesting a high rate of co-morbidity between the two conditions [30–32], and perhaps increased liability for OUD development. Moreover, pain catastrophizing has a moderating effect on the relationship between pain intensity and opioid prescription [33], as well as the relationship between pain and short-acting opioid use [28]. As has been found in non-OUD populations, pain catastrophizing is significantly positively correlated with subjective pain perception in populations of individuals being treated with MT, suggesting a potentially important intervention target [30, 34]. To our knowledge, however, the impact that pain catastrophizing might have on treatment outcomes has not been explored.

The present study sought to explore the impact of trait impulsivity and pain catastrophizing on the important outcome of treatment retention for individuals enrolled in methadone treatment for OUD. We expected that both trait impulsivity and pain catastrophizing would be associated with a greater likelihood of MT dropout. Based on the associations between trait impulsivity and heroin use outcomes reported in previous research [17], we hypothesized that higher trait impulsivity would be associated with lower treatment retention (i.e., more impulsive patients would be less likely to stay in MT).

Similarly, based on previous work identifying pain catastrophizing as a risk factor for opioid use [35], we predicted that higher pain catastrophizing would be associated with lower treatment retention (i.e., individuals with high levels of pain catastrophizing would be more likely to use illicit opioids, and less likely to be retained in MT). Based on previous work in which pain catastrophizing moderated relationships between pain and opioid use, we predicted that pain catastrophizing would moderate the relationship between impulsivity and MT retention. Specifically, we predicted that the association between impulsivity and MT retention would be the strongest in people characterized by high levels of both impulsivity and pain catastrophizing.

## Methods

### Study overview

Data were obtained as part of a larger randomized controlled trial [36, 37] employing a behavioral intervention (ClinicalTrials.gov Identifier NCT02941809, registration date: 10/19/2016). This study was approved by The University of Maryland Institutional Review Board (UMB HRPO identifier # HP-00070829) and all procedures followed ethical standards guided by the Belmont Report and the Declaration of Helsinki. All patients completed a formal written informed consent process to participate in the study. Patients were recruited for the study from December 5, 2017, to August 2, 2019.

### Setting

The University of Maryland Addiction Treatment Center (UMATC) served as the study setting. An urban, community-based opioid treatment program located in West Baltimore, Maryland, the clinic is open 6 days a week (excluding holidays). In addition to providing medications for OUD that include methadone, buprenorphine and naltrexone, the clinic provides counseling, psychosocial support, and psychiatric services. Most patients reside within one of five zip codes immediately surrounding the clinic address, and present for treatment through a variety of referral channels or by self-presentation. During the time frame of data collection, approximately 5 new patients were enrolled into methadone treatment per week.

### Patient recruitment

All participants were recruited from a population of treatment-seeking patients presenting to the UMATC clinic with a primary diagnosis of OUD, and who were enrolled in a clinical trial that was conducted between December 5, 2017, and August 2, 2019. Recruitment methods are described in the published protocol for the parent study [36], but briefly: new patients presenting for methadone treatment were approached on their first day of treatment in the clinic and asked if they were interested in hearing information about a compensated research study testing a novel behavioral approach to enhance methadone treatment outcomes. Participants were randomized to one of two groups: either open-label placebo (OLP), wherein a placebo pill was provided as a daily adjunct to their methadone treatment, or Treatment as Usual (TAU; methadone treatment only). All interactions with the study staff were equal between the groups. Following consent, participants were given several experimenter-administered assessments and met with members of the research team a total of 5 times: at baseline (intake day), two weeks (timepoint 2), and one-, two-, and three months (timepoints 3–5) following their intake. The pain catastrophizing assessment (PCS) score was obtained at intake, and the trait impulsivity score was obtained at the two-week meeting time point. The total period of observation was 365 days.

### Eligibility criteria

Inclusion criteria included the following: (1) adult patients aged 18 years or older and (2) newly admitted to the clinic for methadone treatment of opioid use disorder. Exclusion criteria included: (1) pregnancy, (2) recent methadone treatment elsewhere in the preceding three weeks, 3) hospital transfers (patients initiated on methadone in a hospital setting), 4) criminal justice referrals, and 5) planned opioid treatment with anything other than methadone (including buprenorphine and naltrexone). Given that the purpose of this study was to examine psychological factors associated with treatment retention, and our inability to follow post-treatment administrative discharge outcomes, patients also were excluded from this analysis if they had a discharge/dropout from clinic treatment due to (a) transfer to another substance use treatment agency or to a medical or psychiatric facility, (b) administrative discharge for reasons other than absenteeism (e.g., misconduct), (c) completion of intervention or treatment services (i.e., tapered out of MT), (d) incarceration, (e) death, (f) incomplete admission causing them to not receive the behavioral intervention on day 1; or if they had dropped out of the study before timepoint 2 (the measure of trait impulsivity [BIS-11] was given at timepoint 2). Participant data was included in this study if participants met the above criteria, completed the consent process, and were willing to participate in the randomized behavioral intervention.

### Measures

#### Demographic characteristics

Participant characteristic data (sex, race, ethnicity, education, income, and past-12-month employment status) were collected as part of the baseline drug use history and assessment obtained at the baseline meeting. For demographic characterization of the samples, participants self-identified their sex (female, male, other), race (selection of ≥ 1 of the following categories: American Indian or Alaskan Native, Asian, Black or African American, Native Hawaiian or other Pacific Islander, White, other), and ethnicity (Hispanic or Latinx: yes or no).

#### Barratt impulsiveness scale

The Barratt Impulsiveness Scale (BIS-11) [38] is a 30-item self-report assessment of general trait impulsivity. In the BIS-11, participants endorse a response on four-point Likert-type scales (1 = Rarely/Never, 2 = Occasionally, 3 = Often, 4 = Almost Always/Always) to each of 30 items (e.g., “I say things without thinking,” “I am more interested in the present than the future”). In addition to a total impulsivity score, the BIS-11 also assesses three dimensions of trait impulsivity: attentional impulsivity, motor impulsivity, and non-planning impulsivity. The BIS-11 is widely used in research and clinical settings, and elevated scores are typically observed in individuals with disorders that include deficits in impulse control as a component, such as substance abuse [39].

#### Pain catastrophizing scale

The Pain Catastrophizing Scale (PCS) is a 13-item self-report assessment of catastrophizing based on previous painful experiences [40]. The PCS contains response categories ranging from 0 (not at all) to 4 (all the time); participants rate how often they experience each of 13 thoughts and emotions when they are in pain (e.g., “It’s awful and I feel that it overwhelms me,” “I keep thinking about how badly I want the pain to stop”). The PCS yields an overall pain catastrophizing score, as well as three sub-scales (rumination, magnification, and helplessness). Individual PCS scores of 30 and above were categorized as “high,” based on Sullivan and colleagues’ suggestion that total PCS scores above 30 indicate clinically meaningful levels of pain catastrophizing [40].

### Retention in treatment

Retention in treatment was treated as a dichotomous outcome (1/0) based on whether a participant had dropped out of treatment at any point in time or if they missed 30 consecutive days in a row. In March 2020, the COVID-19 pandemic caused an abrupt, dramatic change to the structure of federal regulations governing methadone treatment [41, 42]. This policy change, implemented to alleviate the density of individuals presenting for daily treatment at busy opioid treatment programs (OTPs) nationwide, effectively lifted pre-pandemic time-in-treatment and adherence requirements to allow a large proportion of our patients to receive up to 28 days of take-home methadone. This change in daily dosing requirements precluded our ability to implement a uniform method of retention analysis. Thus, for purposes of this study, individual data points were right-censored (i.e., the event of dropout had not occurred by the end of the observation period of 365 days) if the participant was still active in treatment as of January 30, 2020 (COVID-19-censored), or if the participant had completed 365 days in treatment.

### Data analysis approach

Frequencies and proportions are reported for discrete variables and means and standard deviations are reported for continuous data. A reliability (internal consistency) assessment was conducted for each of the two measures of pain catastrophizing and impulsivity using Cronbach’s alpha coefficient. We examined retention using a time to event or survival model to predict treatment dropout. We conducted the survival analysis using Cox regression, which predicts the hazard or the probability of surviving the current interval given that one has survived the previous intervals. After estimating the survival function, we used Cox regression to examine correlates of treatment retention. Level of trait impulsivity and level of pain catastrophizing (PCS) were included in all models (i.e., Models One, Two and Three) as continuous covariates. In Model Two, the interaction between trait impulsivity and pain catastrophizing was included as a continuous covariate. Well-powered studies of sufficient sample size have consistently identified younger age as a risk factor for treatment discontinuation [8, 43]. Further, randomized control trial (RCT) group assignment could have had an impact on the observed outcomes. Thus, we incorporated a third model to include age as a continuous covariate and RCT treatment group assignment (i.e., OLP versus treatment as usual [TAU]) as a dichotomous, time-invariant covariate. All analyses were conducted using SPSS v26, and an alpha threshold of statistical significance was set at < 0.05.

## Results

### Demographic characteristics

**Fig. 1:**
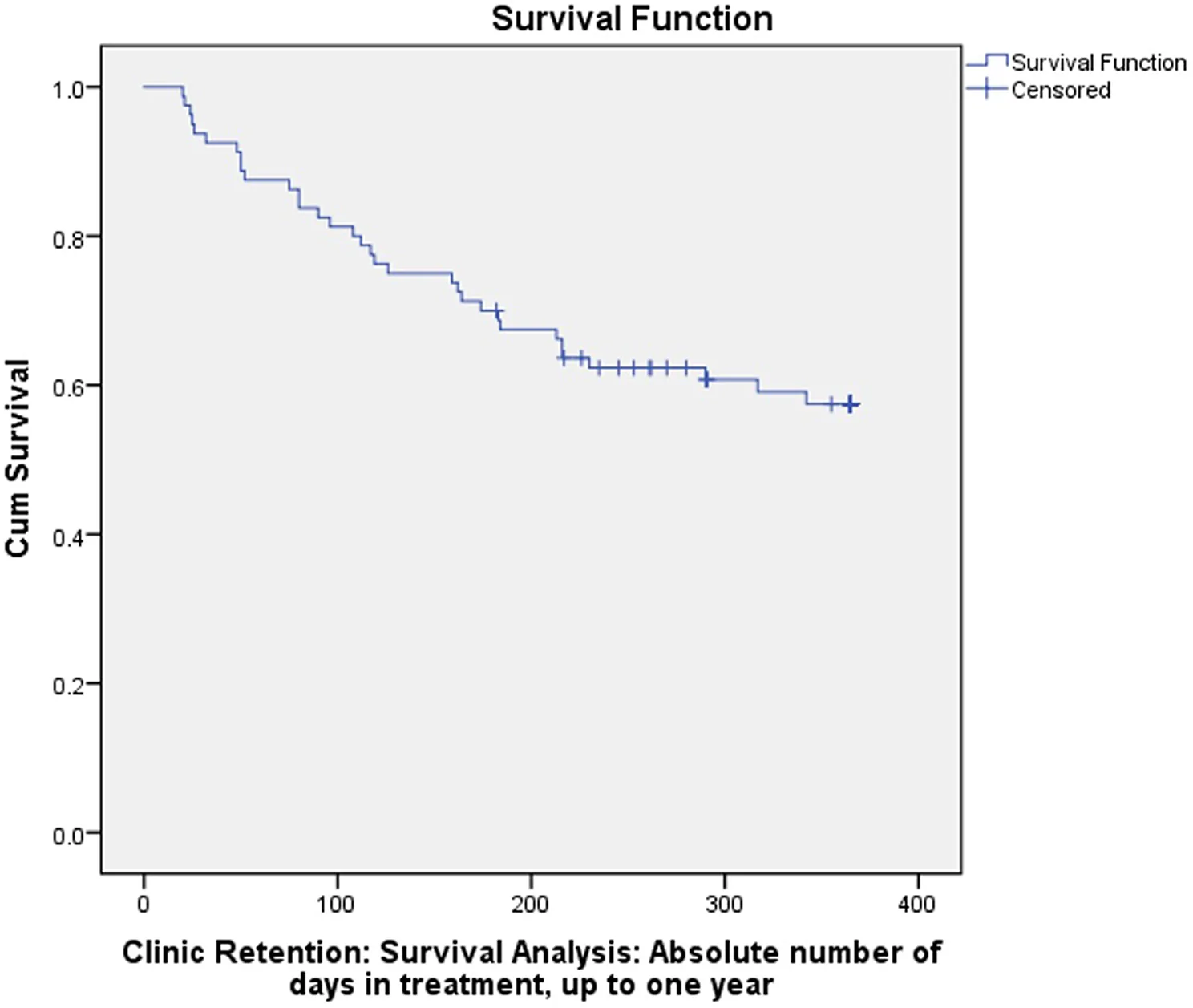
Kaplan-Meier curve depicting time to dropping out of treatment

A total of 320 people were assessed for study involvement, and 187 either did not fit eligibility screening criteria or declined to participate, yielding a total of 133 participants (see Fig. 1 CONSORT flow diagram from Belcher et al., 2023 [37]). Among these 133 individuals, 53 were excluded from these analyses for the following reasons: transfer to another substance use treatment agency (*N* = 3), transfer to a medical or psychiatric facility (*N* = 1), administrative discharge for reasons other than absenteeism (e.g., misconduct) (*N* = 2), completion of intervention or treatment services (i.e., tapered out of MT; *N* = 1), incarceration (*N* = 3), incomplete admission causing them to not receive the behavioral intervention on day 1 (*N* = 1), or treatment drop-out before timepoint 2, and prior to the receipt of the measure of impulsivity (*N* = 42). Thus, a total of 80 participants were included in this analysis as a subset of the larger RCT of participants who had been enrolled in the parent study [37]. As shown in Table 1, the mean age in our study was 47.2 years, and 61% of the participants in our study were male. 70% of the participants identified as African American/Black and 30% as Caucasian/White; none of the participants in our study self-identified as Hispanic. One-third of the sample did not complete high school, and almost half of the participants (49%) reported being currently unemployed. With respect to income, 84% of participants reported earning less than $15,000 in the past year. With respect to the psychological measures collected, the mean impulsivity score was 69.81 (standard deviation [S.D.] = 9.71, and the mean pain catastrophizing scale score was 32.21 (S.D.=13.88; See Table 1). Both measures had good internal reliability, with Cronbach alphas values that were above the conventional standard for acceptability of α>0.70 [44]: BIS-11 α=0.789; PCS α=0.938. Trait impulsivity was positively correlated with pain catastrophizing (*r*[78] = 0.23, *p* = .036).

**Table 1:** Participant demographic characteristics and psychological factors (*N* = 80; percentages rounded up to nearest whole number)

Characteristic	*N* (%)
**Sex**	
Male	49 (61%)
Female	31 (39%)
**Age**	
Mean (S.D.), years	47.2 (10.8)
**Race**	
Black/African American	56 (70%)
White	24 (30%)
**Ethnicity**	
Non-Latino/a	80 (100)
**Highest Education Level Completed**	
Less than high school	26 (33%)
High school graduate	26 (33%)
GED	14 (17%)
Some college	10 (12%)
Associate’s degree	3 (4%)
Bachelor’s degree	1 (1%)
**Income Past 12 Months (%)**	
Less than $15,000	67 (84%)
$15,000 to $19,999	8 (10%)
$20,000+	5 (6%)
**Employment Status Past 12 Months**	
Unemployed	37 (46%)
Retired/disabled	21 (26%)
Homemaker/family caregiver	7 (9%)
Employed part-time	6 (8%)
Employed full-time	6 (8%)
In military service	1 (1%)
In jail/prison	1 (1%)
**Pain Catastrophizing Scale (PCS) Total Score**	
Mean (Standard Deviation [S.D.])	32.21 (13.88)
**Barratt Impulsiveness Scale-11 (BIS-11) Total Score**	
Mean (S.D.)	69.81 (9.71)

### Estimated survival function

The estimated survival function is depicted using a Kaplan-Meier Curve (Fig. 1). Of the 80 participants in this analysis, 47 were censored for the survival analysis and 33 were uncensored.

Of the 47 censored patients, 35 participants were retained in treatment for at least 365 days, and 12 patients were COVID-19-censored. Of the 12 COVID-censored patients, up until January 30, 2020, all 12 made it to at least 6 months in treatment, and only 3 made it to at least 9 months in treatment. Of the 33 uncensored patients, 5 had dropped out by the 1-month timepoint, 13 had dropped out by the 3-month timepoint and 24 had dropped-out by the 6-month timepoint, for a total cumulative one-year survival rate of 58%.

### Main findings

#### Trait impulsivity predicted lower treatment retention

Model 1 included trait impulsivity and pain catastrophizing as independent variables (See Table 2). In this model, higher impulsivity was associated with a higher risk of dropping out of methadone treatment (i.e., higher levels of impulsivity increased the hazard ratio of dropping out of treatment). The effect of pain catastrophizing on treatment retention was not significant in Model 1.

**Table 2:** Pain Catastrophizing, Trait Impulsivity, and the Hazard Ratio (HR) of Dropping Out of Methadone Treatment (Cox Regression)

	Model 1		Model 2		Model 3		
	Hazard Ratio (SE)	CI (95%) Lower Upper	Hazard Ratio (SE)	CI (95%) Lower Upper	Hazard Ratio (SE)	CI (95%) Lower Upper	
**PCS**	0.991 (0.013)	0.966 1.017	1.409 (0.131)**	1.091 1.820	1.364 (0.139)*	1.039	1.789
**BIS-11**	1.040 (0.019)*	1.001 1.080	1.217 (0.064)**	1.074 1.379	1.200 (0.066)**	1.054	1.367
**PCS x BIS-11**			0.995 (0.002)**	0.992.999	0.996 (0.002)*	0.992	0.999
**Age**					0.949 (0.016)**	0.919	0.980
**Treatment Group**							
TAU					1.000		
OLP					0.964 (0.362)	0.474	1.957
N	80		80		80		

*p<.05, **p<.01, ***p<.001

PCS: Pain Catastrophizing Scale Total; BIS-11: Barratt Impulsiveness Scale Total; TAU: Treatment as Usual; OLP: Open Label Placebo

#### Pain catastrophizing moderated the effect of trait impulsivity on treatment retention

Pain catastrophizing moderated the negative impact of trait impulsivity on treatment retention, which was revealed when we added the interaction between trait impulsivity and pain catastrophizing to the model (Model 2, Table 2). Specifically, the effect of impulsivity on treatment retention was greater for people with low levels of pain catastrophizing and was relatively diminished for people with higher levels of pain catastrophizing. In other words, the effect of trait impulsivity on treatment retention increased as level of pain catastrophizing decreased.

**Fig. 2:**
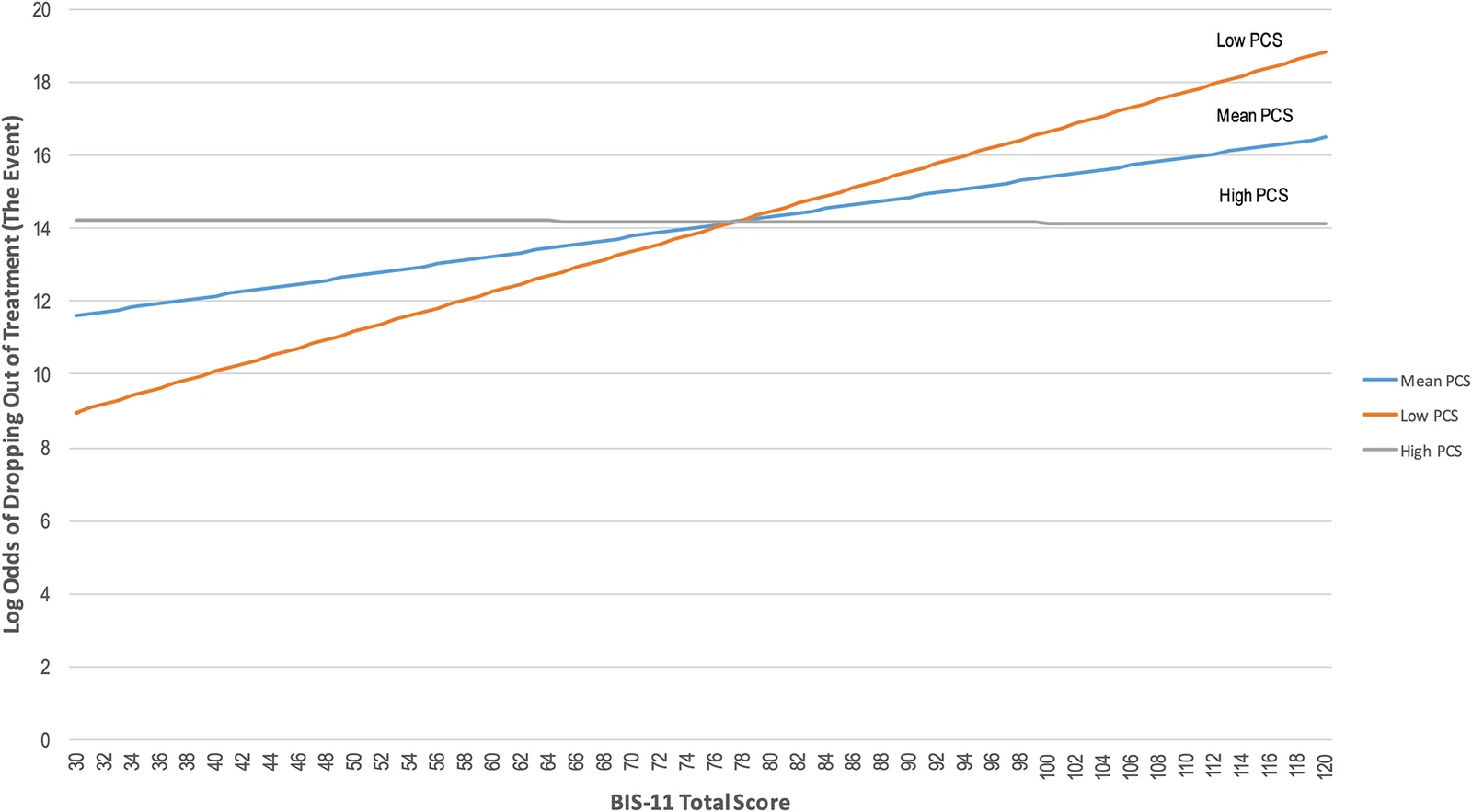
Interaction between impulsivity and pain catastrophizing on treatment drop out

To further illustrate the differential effect of the moderating variable pain catastrophizing, we selected and plotted regression lines for three values of catastrophizing: the mean value and the values one standard deviation below and above the mean (Fig. 2). For subjects with a low PCS score, the odds of dropping out increased by 11.59%. These odds decreased to 5.56% for those subjects with a mean PCS score and decreased further to 0.14% for those subjects with a high PCS score.

#### The relationship of age with treatment retention

Considering previous work demonstrating a positive association between older age and treatment retention [8, 43], we included age, as well as treatment group, in Model 3. Treatment retention was associated with age in our study, with older participants being less likely to drop out (5% per additional year). Treatment group (i.e., OLP versus TAU) had no effect on retention in this study (Model 3, Table 2). Our reported effects remained statistically significant, even after controlling for age and treatment group.

## Discussion

Compelling evidence supports the use of medications (and particularly, treatment with opioid agonists) as the most promising treatment available for OUD. However, the low rate of patient retention is the greatest impediment to individual treatment success. Considering the important role that psychological factors have been shown to play in the determination of substance use disorder (SUD) development [45], it is plausible that some of these factors may also impact SUD treatment outcomes. In this prospective study, we examined whether trait impulsivity and pain catastrophizing predict MT treatment retention in patients with OUD. We expected that trait impulsivity and pain catastrophizing would each predict lower treatment retention. Supporting our first hypothesis, trait impulsivity was associated with retention; higher levels of impulsivity correlated with lower treatment retention. We further report that the effect of impulsivity was enhanced in people with low levels of pain catastrophizing and diminished in people with higher levels of pain catastrophizing. We discuss the main findings below.

Our finding that higher trait impulsivity predicted lower MT treatment retention is consistent with a large body of research identifying trait impulsivity as a risk factor for SUD in general and OUD, in particular [19, 46, 47]. To our knowledge, we are the first to report that higher trait impulsivity was associated with a greater risk of dropping out of methadone treatment (i.e., a lower likelihood of MT treatment retention). Our findings are consistent with previous work reporting that individuals who use heroin are characterized by high levels of trait impulsivity, that trait impulsivity scales with many indicators of addiction severity (i.e., steeper escalation of heroin use, greater overall heroin consumption, greater withdrawal severity), and that trait impulsivity is associated with less treatment-seeking [17–19]. Although the impact of trait impulsivity on clinically relevant MT outcomes has received little attention in the literature, others have observed higher impulsivity in methadone-treated OUD patients, relative to healthy non-opioid dependent controls [19, 48]. Additionally, a large prospective study following OUD-diagnosed individuals entering methadone or buprenorphine treatment found that among other factors, lower trait impulsivity (also assessed using the Barratt Impulsiveness Scale) was positively associated with five-year abstinence [47]. Our finding that higher trait impulsivity predicted lower MT retention is consistent with the definition of trait impulsivity, as a predisposition toward rapid, unplanned actions, with little regard for the negative future consequences. It is possible that highly impulsive people with OUD may be more likely to drop out of MT because not only are they disinclined to consider the consequences of treatment dropout (e.g., withdrawal, relapse, increased risk of overdose), but their tendency toward rapid action also may preclude their opportunity to do so. Our study provides evidence that higher trait impulsivity prospectively predicts lower likelihood of treatment retention—identifying trait impulsivity as a risk factor for low MT retention.

Second, we report that although pain catastrophizing in and of itself did not predict treatment retention, pain catastrophizing interacted with trait impulsivity to impact MT retention. The effect of trait impulsivity on MT retention was greater in people with lower levels of pain catastrophizing and diminished in people with higher levels of pain catastrophizing. The inverse correlation between impulsivity and MT retention was strongest in individuals who also are characterized by *low* pain catastrophizing. It is possible that the correlation between trait impulsivity and MT retention was *weakest* in people who were characterized by *high* pain catastrophizing because the extreme threat of anticipated pain related to opioid-withdrawal MT retention may have dampened the likelihood of MT dropout in those individuals, regardless of level of trait impulsivity. This finding suggests that examining the interaction of pain catastrophizing with trait impulsivity may yield greater predictive value over that of impulsivity alone. Our study therefore provides a more nuanced understanding of the relationship between trait impulsivity and MT retention.

Previous work in acute and chronic pain patients demonstrated that patients with high levels of pain catastrophizing experience more intense pain [24, 26], have more severe depression and anxiety [24, 25], show higher levels of pain behavior and disability [27, 47–52], and consume more analgesic medication [28, 53, 54]. As described earlier, pain catastrophizing has been closely tied to using larger amounts of prescription opioids [28], higher levels of opioid craving [29], and greater rates of opioid prescription misuse [35]—studies that collectively suggest an increased liability for development of OUD. In the few studies with methadone-treated OUD patients, pain catastrophizing was associated with greater pain severity, pain interference, and sleep disturbance—however, none of the studies examined whether pain catastrophizing was associated with MT outcomes [30, 34, 55, 56]. We report that pain catastrophizing moderated the association between trait impulsivity and greater risk of MT dropout, such that the effect of trait impulsivity on likelihood of treatment dropout was greatest in individuals who also are characterized by *low* pain catastrophizing. This finding underscores the orthogonality of these two psychological factors and identifies a specific combination of traits that may confer an increased risk of low MT retention. Additionally, the finding that older age was a significant predictor of greater treatment retention is consistent with previous work [8, 43, 57]. Our findings demonstrate the added value of psychological and personality factors in predicting methadone treatment retention, even after controlling for age.

Our findings also have clinical utility; understanding how patient personality traits and characteristics impact MT retention could guide within-clinic targeted treatment approaches. Self-report assessments of trait impulsivity and pain catastrophizing are brief and would be relatively easy to administer in a clinical setting. It is possible that measuring MT patients’ levels of trait impulsivity and pain catastrophizing would be useful to help identify patients with an especially high risk of treatment dropout, so that such patients might receive additional support services tailored to addressing and modifying impulsive behaviors [58]. Impulsivity spans many disorders outside of addictions and has been shown to be ameliorated with targeted structured psychotherapies (predominantly, Dialectical/Cognitive Behavior Therapy [DBT/CBT]), as well as cognitive training approaches (e.g., Goal Management Training) [59]. Purposeful early clinical attention to personality and psychological constructs that confer vulnerability and/or risk could help improve addiction treatment outcomes [58, 60].

### Limitations and conclusions

The present findings provide preliminary insights into personality and psychological factors that contribute to MT retention outcomes. Our study is not without limitations. First, because trait impulsivity was assessed two weeks after intake, we were unable to assess those patients who dropped out before that time. Second, this exploratory study was not statistically powered to examine the impact of psychological factors on treatment retention. Additional studies with larger samples are needed to assess the reproducibility of these findings.

Additionally, as a single-site trial that was conducted in a busy OTP in Baltimore, Maryland, these findings may not generalize to other areas or OTP settings, which may have fewer resources than those found in an urban setting. Finally, we necessarily excluded some groups of patients from participation (i.e., patients who were mandated to enter treatment by the criminal-legal system, etc.); a fact that may render a less accurate capture of the broader patient population, further limiting generalizability. Researchers wishing to extrapolate the current findings to other settings and populations should consider these generalizability limitations.

In conclusion, our findings suggest that higher trait impulsivity predicts lower retention in MT. Further, the effect of trait impulsivity on retention was moderated by pain catastrophizing in our study, such that the effect was greatest in people with the lowest levels of catastrophizing. These novel findings advance our understanding of trait impulsivity and pain catastrophizing and contribute to the growing body of literature on the relationship of psychological factors to treatment outcomes for patients with SUD.

## Data Availability

Researchers wishing to access the data may submit a statement detailing the proposed use of the data to the corresponding author: abelcher@som.umaryland.edu.

## Abbreviations

BIS-11: Barratt Impulsiveness Scale
CI: Confidence interval
MT: Methadone treatment
OLP: Open-label placebo
OUD: Opioid use disorder
PCS: Pain catastrophizing assessment
RCT: Randomized control trial
SUD: Substance use disorder
TAU: Treatment as usual
UMATC: University of Maryland Addiction Treatment Center
